# May-Thurner Syndrome: A Rare Cause of Deep Venous Thrombosis

**DOI:** 10.7759/cureus.2700

**Published:** 2018-05-29

**Authors:** Sidra Khalid, Aariez Khalid, Tessy Meridores, Hamed Daw

**Affiliations:** 1 Internal Medicine Residency, Fairview Hospital, Cleveland Clinic, Cleveland, USA; 2 Biomedical Science, University of Guelph, Guelph, CAN; 3 Internal Medicine, Fairview Hospital, Cleveland Clinic, Cleveland, USA; 4 Department of Hematology and Oncology, Fairview Hospital, Cleveland Clinic, Cleveland, USA

**Keywords:** anticoagulation, stent, may thurner syndrome

## Abstract

May-Thurner syndrome (MTS) is a medical condition where the left iliac vein is compressed by the right iliac artery, which in turn predisposes patients to deep venous thrombosis (DVT). We present a case of a 67-year-old female who had pain and swelling of the left leg. Ultrasound of the deep veins of the leg revealed DVT of the distal external iliac vein. She was treated with catheter-directed thrombolysis and stent placement. Finally, she was discharged on long-term anticoagulation with warfarin. The purpose of presenting this case is to highlight the clinical presentation, diagnosis, and treatment of MTS.

## Introduction

May-Thurner syndrome (MTS) is a condition in which the left iliac vein is compressed between the overlying right iliac artery and the lumbar spine. This anatomic variant has a prevalence of about 22%-24% [1]. It typically presents as left lower extremity edema and pain. Diagnostic modalities help in detecting deep venous thrombosis (DVT) and the anatomic characteristic of the syndrome, it also helps rule out a pelvic mass. Since it presents as a DVT, it is important to manage this condition as it can result in pulmonary embolism leading to morbidity and mortality. Treatment involves thrombolysis, stent placement, and long-term anticoagulation. Long-term anticoagulation is especially indicated in patients who have a stent in place.

## Case presentation

**Figure 1:**
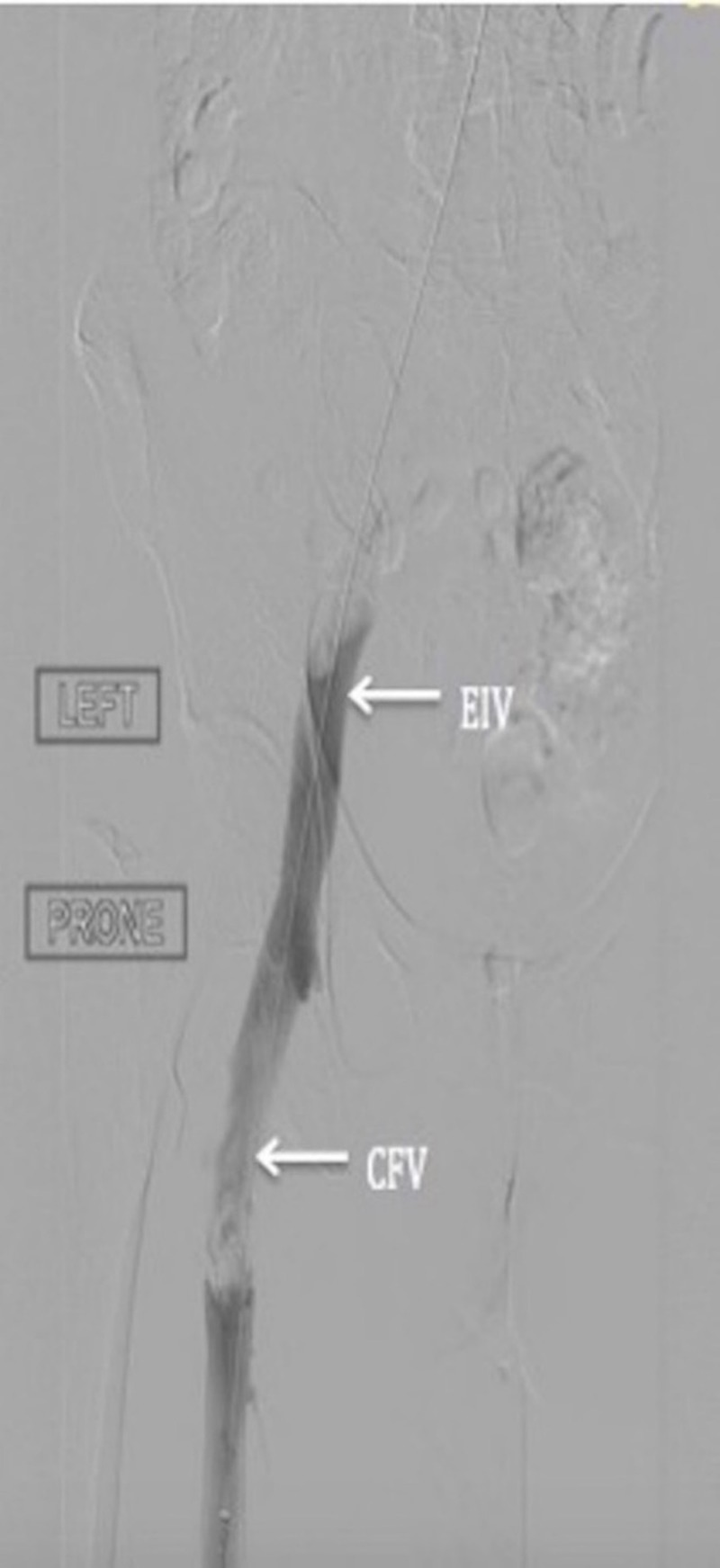
Angiogram of left lower extremity veins; irregularity suggestive of thrombus in the external iliac vein (EIV) and common femoral vein (CFV)

A 67-year-old female presented to the emergency department with worsening left leg pain and swelling for two days. On presentation, her vital signs and physical examination were unremarkable except for extensive edema of the left leg. DVT ultrasound of the lower extremities revealed an acute DVT of the left distal external iliac, common, and superficial femoral veins. Heparin infusion was initiated. Since the clot was large and recent, thrombolytic therapy was planned. Through ultrasound guided cannulation of the left popliteal vein, catheterization of the inferior vena cava (IVC) from the popliteal vein was performed. A left leg and abdominal venogram showed a patent dilated superficial femoral vein, with a dense thrombus involving the proximal superficial femoral vein extending into the common femoral, external, and common iliac veins (Figure 1).

**Figure 2:**
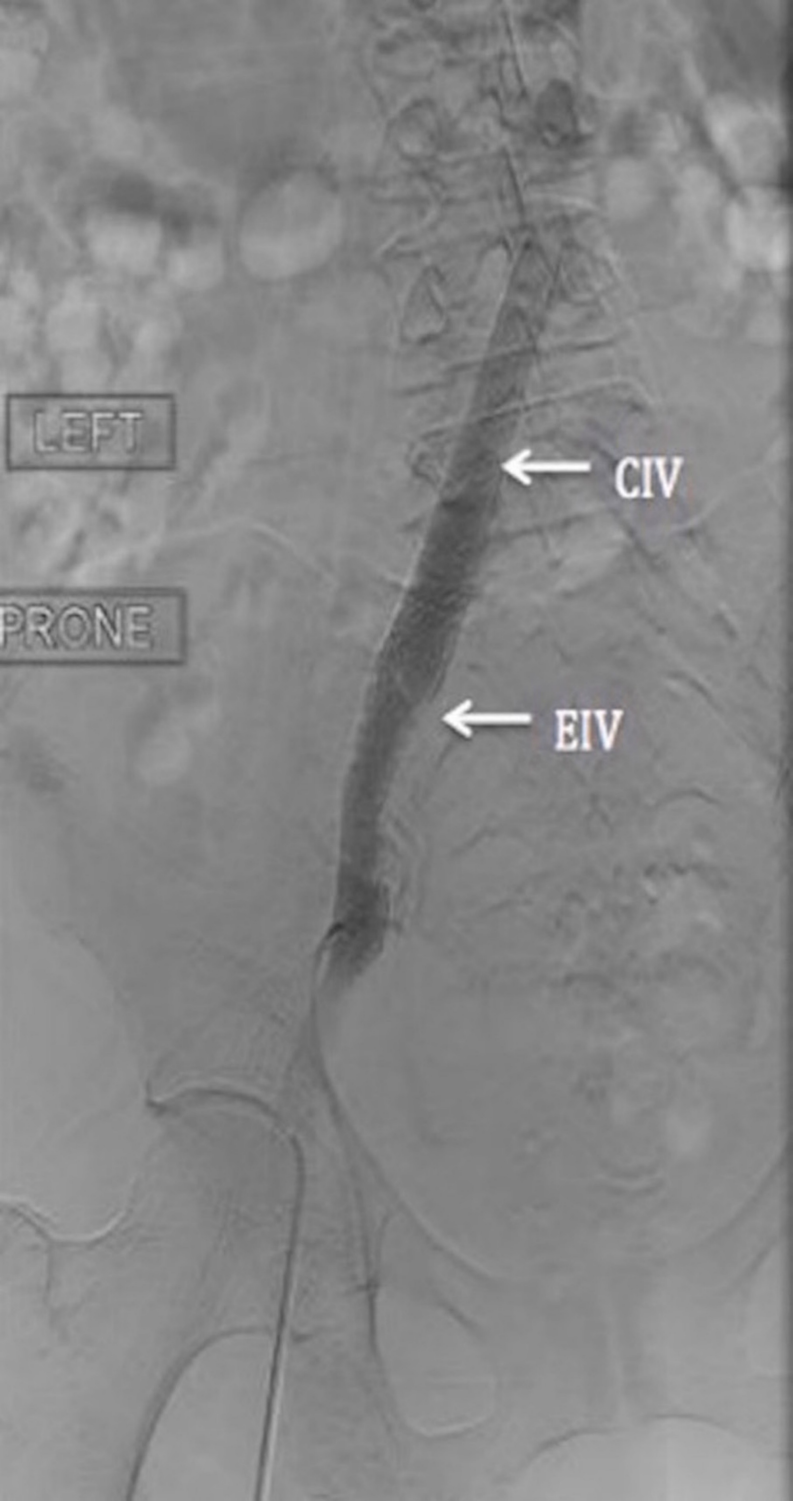
Stents placed in the common iliac vein (CIV) and external iliac vein (EIV)

A dose of 12 mg alteplase was infused into the thrombus. Subsequently, catheter directed thrombolysis was perfomed. This led to a resolution of thrombus in the superficial and common femoral veins, but there was significant residual stenosis and thrombus in the left common and external iliac veins. Angioplasty of the left common and external iliac, superficial and common femoral veins was performed. Catheter directed therapy with alteplase at 0.5 mg/hr was infused overnight. The following day, angiography showed patency of the left femoral and external iliac veins, but no forward flow in the left common iliac vein. Intravascular ultrasound (IVUS) revealed a residual thrombus and extrinsic compression of the left common iliac vein from the crossing artery. A diagnosis of MTS was made. Stents were placed in the common and external iliac veins, after which there was no residual irregularity, and a forward flow into the IVC was achieved (Figure 2). She was discharged on long-term oral anticoagulation therapy with warfarin.

## Discussion

MTS is a medical condition that affects 18%-49% of the patients who have DVT of the left lower extremity. MTS is a risk factor for developing left sided iliofemoral DVT. There are many postulated mechanisms that cause MTS. Firstly, it occurs due to chronic pulsations of the overriding right iliac artery which leads to the development of a spur in the wall of the vein. This spur then causes obstruction of the region leading to clot formation. Secondly, due to the chronic pulsations of the overlying artery, there is also trauma to the venous walls, which leads to deposition of collagen and elastin that impairs venous return leading to DVT. Thirdly, hypercoagulable states also contribute to DVT. Patients with this condition usually present with the left lower extremity edema and pain. Due to the chronicity of the DVT, patients also present with pigment changes, varicose veins, chronic leg pain, and phlebitis. These conditions usually have increased incidence in young women in their twenties to forties, especially after pregnancy and prolonged immobilization [2].

Diagnosis depends on the clinical presentation of left lower extremity pain and swelling. Doppler ultrasound could be useful to detect the DVT; however, it is unable to visualize the iliac vein compression and the location of the spur. Other modalities include helical abdominal computed tomography (CT), CT venography, magnetic resonance venography, IVUS, and conventional venography [2]. In a CT scan, the pelvic CT images in the transverse plane help detect iliac vein compression by the overlying common iliac artery [3]. Magnetic resonance imaging (MRI) is a useful modality as it can detect the presence of a pelvic mass, DVT, and demonstrate the anatomic characteristics of the syndrome [4]. The gold standard for diagnosing MTS is conventional venography [2].

Treatment for MTS involves clearing the thrombus and correcting the compression of the left iliac vein. Endovascular intervention with thrombolysis and stenting is considered the first line treatment for MTS. Catheter-directed thrombolysis could be performed, in which urokinase or tissue plasminogen activator (tPA-alteplase) is administered locally at the site of the thrombus. It is very effective in reducing the clot burden. Also, mechanical thrombectomy can be performed to reduce the thrombolytic infusion time and/or complications. After thrombectomy or thrombolysis, angioplasty with stent placement is done to relieve the obstruction [2]. Moudgill et al. studied 113 patients with left-sided extensive DVT who were treated with catheter-directed thrombolysis and stent placement. Their results showed a mean technical success of 95% and a mean one-year patency of 95% [5]. In a study by Hager et al., stenting for MTS, for patients with postthrombotic and edema alone, was considered safe, effective and durable for 36 months, with a patency rate of 91% [6]. If endovascular treatment alone is unsuccessful, then stent implantation could be performed surgically. Igari et al. performed venous thrombectomy with simultaneous stent placement to treat DVT with MTS, which resulted in venous patency and relief of symptoms [7]. Subsequently, after clearing the thrombus and placing a stent, patients are routinely treated with anticoagulation to maintain venous patency and prevent re-stenosis of the stent [2]. Additionally, after placing a stent there is a debate about the duration of anticoagulation. Anticoagulation should be continued for six months and then the patient’s risk of DVT is reassessed to decide the duration of anticoagulation [8]. At the time of reassessment, the factors to consider are the presence of a venous stent, underlying etiology of the DVT, patient preference, patient compliance, bleeding risks, co-morbidities, polypharmacy, and risk of recurrence [9].

In our case, the patient underwent thrombolysis and stent placement. Finally, long-term anticoagulation therapy was given to prevent the relapse. Therefore, a multidisciplinary approach, as indicated in our case, is important to manage this condition effectively.

## Conclusions

Our case highlights that MTS is a rare condition, which if diagnosed early can be effectively managed with thrombolysis, stent placement, and long-term anticoagulation.
